# Commercially available CD4 + and CD8 + IFN-γ release assays combined with an HBHA-induced IGRA improve the characterization of the tuberculosis spectrum and monitoring of treatment in children

**DOI:** 10.1007/s00431-023-04844-1

**Published:** 2023-02-27

**Authors:** Danilo Buonsenso, Giovanni Delogu, Maria del Carmen Pereyra Boza, Flavio De Maio, Ivana Palucci, Laura Martino, Davide Pata, Maurizio Sanguinetti, Piero Valentini, Michela Sali

**Affiliations:** 1 Department of Woman and Child Health and Public Health, Fondazione Policlinico Universitario A. Gemelli IRCCS, Largo A. Gemelli 8, 00168 Rome, Italy; 2 Global Health Research Institute, Istituto di Igiene, Università Cattolica del Sacro Cuore, Rome, Italy; 3 Dipartimento di Scienze di Laboratorio e infettivologiche, Fondazione Policlinico Universitario A. Gemelli IRCCS, Rome, Italy; 4 Dipartimento di Scienze biotecnologiche di base, cliniche intensivologiche e perioperatorie – Sezione di Microbiologia, Università Cattolica del S. Cuore, Milan, Italy

**Keywords:** Tuberculosis, Children, Biomarkers, IGRA, HBHA

## Abstract

Commercially available Interferon-γ release assays (IGRAs), including the last-generation QuantiFERON TB-Plus (QFT-Plus), are effective in aiding the diagnosis of tuberculosis (TB) infection but cannot distinguish latent TB subjects from active TB patients. The aim of this study was to prospectively evaluate the performance of an HBHA-based IGRA, combined with commercially available IGRAs, to assess their usefulness as a prognostic biomarkers and aid in the monitoring of TB treatment in children. Following clinical, microbiological, and radiological assessment, children younger than 18 years of age classified as either LTBI or active TB were tested at baseline and during treatment by the QuantiFERON TB-Plus (QFT) assay and an aliquot of whole-blood was stimulated with HBHA. Among the 655 children evaluated, 559 (85.3%) were classified as “Non TB”, 44 patients (6.7%) with active TB, and 52 (7.9%) with LTBI. The median HBHA-IGRA IFN-gamma responses were able to discriminate active TB from LTBI (0.13 IU/ml vs 1.995, (*p* < 0,0001), those with asymptomatic TB from those with symptomatic TB (1.01 IU/ml vs 0.115 IU/ml, *p* 0.017), or more severe TB (p 0.022), and significantly raised during successful TB treatment (*p* < 0.0001). Conversely, CD4 + and CD8 + responses were similar in all groups of patients, although active TB patients had higher CD4 + responses and LTBI higher CD8 + responses.

*  Conclusion*: HBHA-based IGRA, combined with CD4 + and CD8 + responses assessed by commercially available IGRAs, is a useful support in the characterization of the TB spectrum in children and monitoring of TB-therapy.

**Table:** 

**What is Known:** *• Current immune diagnostics are not able to discriminate active and latent Ttuberculosis, including the recently approved QFT-PLUS.*. *• New immunological assays with prognostic value are highly needed*.	
**What is New:** *• HBHA-based IGRA, combined with CD4*+ *and CD8*+ *responses assessed by commercially available IGRAs, is a useful support for the differentiation of active and latent TB in children.*. *• HBHA-based IGRA, combined with CD4*+ *and CD8*+ *responses assessed by commercially available IGRAs, is a useful support in the monitoring of TBtherapy in children.*.

**What is Known:**

*• Current immune diagnostics are not able to discriminate active and latent Ttuberculosis, including the recently approved QFT-PLUS.*.

*• New immunological assays with prognostic value are highly needed*.

**What is New:**

*• HBHA-based IGRA, combined with CD4*+ *and CD8*+ *responses assessed by commercially available IGRAs, is a useful support for the differentiation of active and latent TB in children.*.

*• HBHA-based IGRA, combined with CD4*+ *and CD8*+ *responses assessed by commercially available IGRAs, is a useful support in the monitoring of TBtherapy in children.*.

## Introduction

Despite the significant improvements in tuberculosis (TB) control programs of the last decades, pediatric TB remains relatively neglected, mainly due to the greater challenges in its diagnosis and microbiological confirmation, and for its lower contribution in community transmission [1]. Nevertheless, in 2019 1.2 million children fell ill with TB globally, representing 10–11% of all TB cases and, according to the last World Health Organization (WHO) reports, approximately 230,000 children die of TB every year [2]. Moreover, the COVID-19 pandemic had a negative impact on the TB screening programs and global studies estimated that several opportunities for early diagnoses of disease and latent TB infection (LTBI) have been missed, raising concerns for a new surge of cases in the coming years [3, 4].

Diagnosis of childhood TB faces several challenges mainly due to the following: (i) the lack of specific signs and symptoms consistent with TB; (ii) the limited amounts of sputum produced by children; (iii) the paucibacillary nature of TB disease in children compared to adults [5]. Additionally, early detection and treatment of TB infection (TBI) plays a fundamental role in reducing the risk of developing active disease. In fact, children are at much higher risk of progression to active disease than adults, with 30–40% of children having TB infection progressing to active TB disease and 10–20% of TB infected children developing pulmonary disease within the second year of life [6, 7]. Therefore, immunological tests with clinical prognostic value would improve diagnosis of TB in children and contribute to achieve the TB elimination goals [1, 2].

The most widely used immunological tests, such as the tuberculin skin test (TST) and the first-generation Interferon-gamma release assays (IGRAs, such as the QuantiFERON-TB Gold in Tube (QFT-GIT) and the T-SPOT.TB), although useful and effective in detecting specific immune responses, cannot distinguish within the complex and wide spectrum of TB infection and such these assay do not have prognostic value [8–12]. For these reasons, a new-generation IGRA, the QuantiFERON-TB Gold Plus (QFT-Plus) has been developed, adding to the predecessors an additional antigen tube (TB2), containing shorter peptides of ESAT-6 and CFP-10 which can stimulate both CD4 + and CD8 + T cells, and keeping the same TB1 tube of the QFT-GIT that only stimulates CD4 + cells. According to early adult studies, the possibility to discriminate CD4 + and CD8 + responses can add sensitivity and specificity in adults and help discriminate active TB from LTBI [10]. However, early pediatric reports demonstrated that in children also the QFT-Plus is not able to discriminate the more complex spectrum of TB disease [8–13].

Nowadays, the mycobacterial Heparin-binding haemagglutinin (HBHA) is a promising diagnostic latency-associated antigen and several researchers are evaluating the ability of HBHA-induced IFN-gamma release assay in discriminating latent tuberculosis infection from active TB [14, 15]. A previous pediatric study showed that an HBHA-base IGRA in combination with QFT-GIT improved the ability to discriminate active TB from LTBI in children [16]. A combined use of QFT-Plus and HBHA-IGRA, discriminating CD4 + and CD8 + responses, may have the potential of distinguishing active TB from LTBI in children. Therefore, we performed this study aiming to evaluate the ability of an HBHA-based IGRA combined with the TB1 and TB2 assays to distinguish active TB cases from Mtb infection and to monitor children’s response to treatments.

## Materials and methods

### Study population

This prospective study was conducted on children younger than 15 years of age evaluated for LTBI or TB disease at the Fondazione Policlinico Universitario A. Gemelli University Hospital, in Rome (Italy). This study is an update of a previously published study where we assessed children using the QFT-GIT [16]. For this study, new patients with suspected TB or LTBI have been tested with QFT-Plus and were classified as “non TB,” healthy children who were screened for LTBI and scored negative to QFT-Plus or children admitted for presumed TB who were eventually diagnosed with any infectious disease other than active TB or LTBI; “LTBI,” children scoring positive to QFT-Plus and showing normal chest radiography in the absence of any clinical/microbiological feature suggestive of active disease [17–19]; and “active TB,” children with signs or symptoms suggestive of active disease and/or at least one clinical specimen scoring positive for Mtb following microbiological analysis [17–19]. Children initially diagnosed with active TB were classified into three categories following the severity of clinical characteristics:

- Asymptomatic active TB: children showing radiological or microbiological evidence of disease, without any signs or symptoms;
- Moderate active disease: children showing signs and symptoms of pulmonary tuberculosis disease;
- Severe active disease: children affected by disseminated forms of disease, including military pattern, tuberculous meningo-encephalitis, osteoarticular localization, or involvement of other organs.

Patients diagnosed with LTBI or active TB were proposed to take an IGRA and HBHA-IGRA at the moment of diagnosis (T0), after 3 months of treatment (T1), and at the end of treatment (T2).

Children with immunodeficiencies (human immunodeficiency virus related or unrelated) were excluded.

### Interferon-gamma release assays

For this study, children assessed before 2016 were tested with QFT-GIT, while since 2016 in our Institution we introduced QFT-Plus (QIAGEN, Germantown, MD, USA). Both tests were performed according to manufacturer’s instructions (REF). The Nil and the Mitogen tubes are the same for both assays, while the QFT-Plus assays include a second antigen tube (TB2) containing shorter ESAT-6 and CFP-10 peptides, aimed at eliciting responses from CD4 + and CD8 + T cells, in addition to the first antigen tube (TB1), which contains peptides capable of eliciting CD4 + T cell responses, as happened with the tube of QFT-GIT [11]. To perform the HBHA-IGRA, an extra aliquot of blood was collected in the Nil Control QFT tube of patients with active TB or LTBI and 5 µg/ml of recombinant methylated HBHA was added and incubation was carried out at 37 °C for 16–24 h. Following incubation, supernatants were collected by centrifugation and IFN-γ concentration determined by a cytokine ELISA (Qiagen, Venlo, The Netherlands). Therefore, we provided results separately for TB1 and TB2 tubes, along with results from the HBHA-IGRA.

Data are presented as IU/ml of IFN-γ. The cut-off value for a positive test was 0.35 IU/ml for QFT (as indicated by the manufacturer), and 0.25 IU/ml for the HBHA-based IGRA, as previously determined in adults [20] and in our previous study [16].

### Purification of recombinant methylated HBHA

HBHA was purified from *M. smegmatis* pMV3-38 as previously described [21] and batches of purified protein were tested for the presence of LPS using Limulus Amebocyte Lysate QCL-1000TM (Lonza Walkersville, MD USA) (LPS < 1 IU/ml).

### Statistical analyses

Data were analyzed using SPSS (SPSS, Chicago, IL) and Prism 5 software (Graphpad Software 5.0, San Diego, CA, USA). Differences in frequencies were evaluated by the Fisher exact test. The median of IFN-γ production was calculated; the non-parametric Mann–Whitney *U* test was used to compare medians for unpaired comparisons and the Wilcoxon test for paired comparisons; the Kruskal–Wallis test was used to compare medians among the different groups of TB and LTBI patients, including the TB subgroups analyzed. Differences were considered significant at *p* values ≤ 0.05.

### Ethics statement

The study was approved by the Ethical Committee of the Catholic University of the Sacred Heart, Rome (“Parere 23,866/13”, UCSC, Rome) and all enrolled individuals provided written informed consent.

### Data availability

Available upon requests to the corresponding authors.

## Results

### Characteristics of the population

**Fig. 1:**
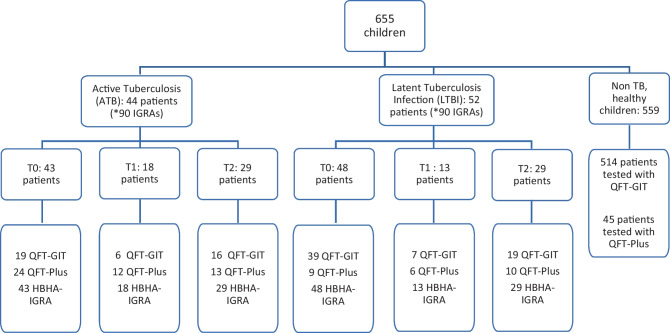
Study population enrolled in the study

A total of 655 children, 378 males (57.7%) and 277 females (42.3%), were evaluated for either suspected (LTBI) or active TB. Following clinical assessment and IGRA test, among the 655 children, 559 (85.3%) were classified as “Non-TB,” healthy children or children diagnosed with any infectious disease other than active TB or LTBI; 44 patients (6.7%) were diagnosed with active TB and 52 (7.9%) with LTBI (Fig. 1). Of the 44 patients with active TB, 8 (18.2%) were asymptomatic, while 36 (81.8%) had signs/symptoms consistent with TB (e.g., cough, fever, neurological or osteoarticular symptoms). Four children had severe TB with extrapulmonary localization (two with central nervous system TB, one with severe Pott disease, one with disseminated TB involving the lungs, pleura, mediastinal lymph nodes, kidneys, and liver).

The median age of the study population was 115 months (range, 3 to 227 months). Most children evaluated came from Eastern Europe (31.5%). Among the children diagnosed with active TB, 36 showed the typical disease symptoms while 8 were asymptomatic. Further demographic characteristics and main QFT and HBHA-IGRA results are detailed in Table 1.

**Table 1:** Demographic and clinical characteristic of the subjects enrolled

		***Total***	***Non TB***	***aTB***	***LTBI***
***N (%)***		655 (100)	559 (85.3)	44 (*90 IGRAs)	52 (*90 IGRAs)
***Gender N (%)***					
	Female	277 (42.3)	236 (42.2)	20 (45.5)	21 (40.4)
***Age (months) N (%)***					
	0–12	27 (4.1)	21 (3.7)	4 (9.1)	2 (3.8)
	13–60	264 (40.3)	238 (42.6)	16 (36.3)	10 (19.2)
	61–120	279 (42.6)	247 (44.2)	6 (13.6)	26 (50.0)
	>120	85 (13.0)	53 (9.5)	18 (41.0)	14 (27.0)
***Nationality N (%)***					
	Eastern Europe	207 (31.5)	168 (30.0)	18 (41.0)	21 (40.4)
	Italy	59 (9.0)	42 (7.5)	6 (13.6)	11 (21.2)
	Western Countries	23 (3.5)	19 (3.4)	2 (4.5)	2 (3.8)
	Africa	117 (18.0)	101 (18.1)	9 (20.4)	7 (13.5)
	Asia	126 (19.2)	115 (20.6)	5 (11.4)	6 (11.5)
	South America	123 (18.8)	114 (20.4)	4 (9.1)	5 (9.6)
***Contact with active TB N (%)***					
	Yes	53 (8.0)	18 (3.2)	21 (47.7)	14 (27.0)
	Unknown	602 (92.0)	541 (96.8)	23 (52.3)	38 (73.0)
***BCG vaccination N (%)***					
	Vaccinated	261 (40.0)	240 (43.0)	6 (13.6)	15 (28.8)
	Unvaccinated	177 (27.0)	130 (23.2)	23 (52.3)	24 (46.2)
	Unknown	217 (33.0)	189 (33.8)	15 (34.1)	13 (25.0)
***QFT-Plus N (%)***		75 (11.4)	45 (8.0)	21 patients (47.7)	9 patients (17.3)
				49 IGRA tests (54.4)	25 IGRA tests (27.8)
	***QFT-Plus Results N (%)***				
	* Positive*	26 (36)	0	18 patients (85.7)	9 patients (100)
				42 IGRA tests (85.7)	25 IGRA tests (100)
	* T0*			18 (75)*	9 (100)
	* T1*			12 (100)	6 (100)
	* T2*			12 (92.3)	10 (100)
	* Negative*	42 (56)	39 (86.7)	3 patients (14.3)	0
				7 IGRA tests (10.6)	
	* T0*			6 (25.0)	0
	* T1*			0 (0)	0
	* T2*			1 (7.7)	0
	* Indeterminate*	6 (8)	6 (13.3)	0	0
***QFT-Gold In Tube N (%)***		580 (88.6)	514 (92)	23 patients (52.2)	43 patients (82.7)
				41 IGRA tests (46.6)	65 IGRA tests (72.2)
	***QFT-Gold In Tube Results N (%)***				
	* Positive*	66 (11.4)	0	23 patients (100)	43 patients (100)
				41 IGRA tests (100)	65 IGRA tests (100)
	* T0*			19 (100) **	39 (100)
	* T1*			6 (100)	7 (100)
	* T2*			16 (100)	19 (100)
	* Negative*	505 (87.0)	505 (98.2)	0	0
	* Indeterminate*	9 (1.55)	9 (1.8)	0	0
***HBHA cut-off 0.25 N (%)***					
	*Positive*		426 (76.2)	57 (63.3)	71 (78.9)
	*T0*			19 (44.2) ***	40 (83.3)
	*T1*			13 (72.2)	8 (61.5)
	*T2*			25 (86.2)	23 (79.3)
	*Negative*		133 (23.8)	33 (36.7)	19 (21.1)
	*T0*			24 (55.8)	8 (16.7)
	*T1*			5 (27.8)	5 (38.5)
	*T2*			4 (13.8)	6 (20.7)
***HBHA cut-off 1.00 N (%)***					
	*Positive*		303 (54.2)	36 (40.0)	52 (57.8)
	*T0*			8 (18.6)****	30 (62.5)
	*T1*			8 (44.4)	5 (38.5)
	*T2*			20 (69.0)	17 (58.6)
	*Negative*		256 (45.8)	54 (60.0)	38 (42.2)
	*T0*			35 (81.4)	18 (37.5)
	*T1*			10 (55.6)	8 (61.5)
	*T2*			9 (31.0)	12 (41.4)

*T0* at the moment of diagnosis, *T1* after 3 months of treatment, *T2* at the end of therapy

*(18/24 = T0 positive QFT Plus/T0 Total QFT-Plus)

**(19/19 = T0 positive QFT-GIT/T0 total QFT-GIT)

***(19/43 = T0 positive HBHA cut-off 0.25/T0 total HBHA cut-off 0.25 tested)

****(8/43 = T0 positive HBHA cut-off 1.00/T0 total HBHA cut-off 1.00 tested)

### QFT and HBHA-IGRA quantitative responses in children with active TB and LTBI

**Fig. 2:**
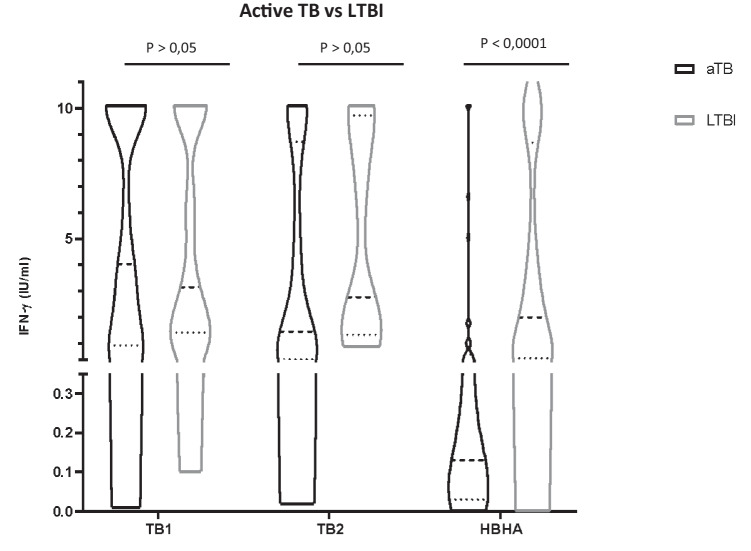
TB1, TB2, and HBHA IFN-gamma responses in children with active TB and LTBI

We compared TB1, TB2, and HBHA responses in children with active TB and LTBI (Fig. 2). Based on the results obtained, QFT could not discriminate active TB from LTBI in children. In particular, TB1 and TB2 responses were similar in each group and did not present statistically significant differences (*p* > 0.05), although TB1 IFN-γ levels were slightly higher in active TB (4.03 IU/ml vs 3.14 IU/ml *p* > 0.05) and TB2 IFN-γ levels were slightly higher in LTBI (2.76 IU/ml vs 1.44 IU/ml, *p* > 0.05). In contrast, the amount of IFN-γ released after stimulation with methylated rHBHA was significantly higher in children diagnosed with LTBI (1.95 IU/ml) compared with those with active TB (0.13 IU/ml, *p* < 0.0001). The HBHA-based IGRA was positive in 47 (90.3%) out of the 52 patients with LTBI, while 18 (41%) of the 44 active TB patients scored HBHA-positive.

### Dynamic changes of TB1, TB2, and HBHA quantitative responses during treatment for active TB and LTBI

**Fig. 3:**
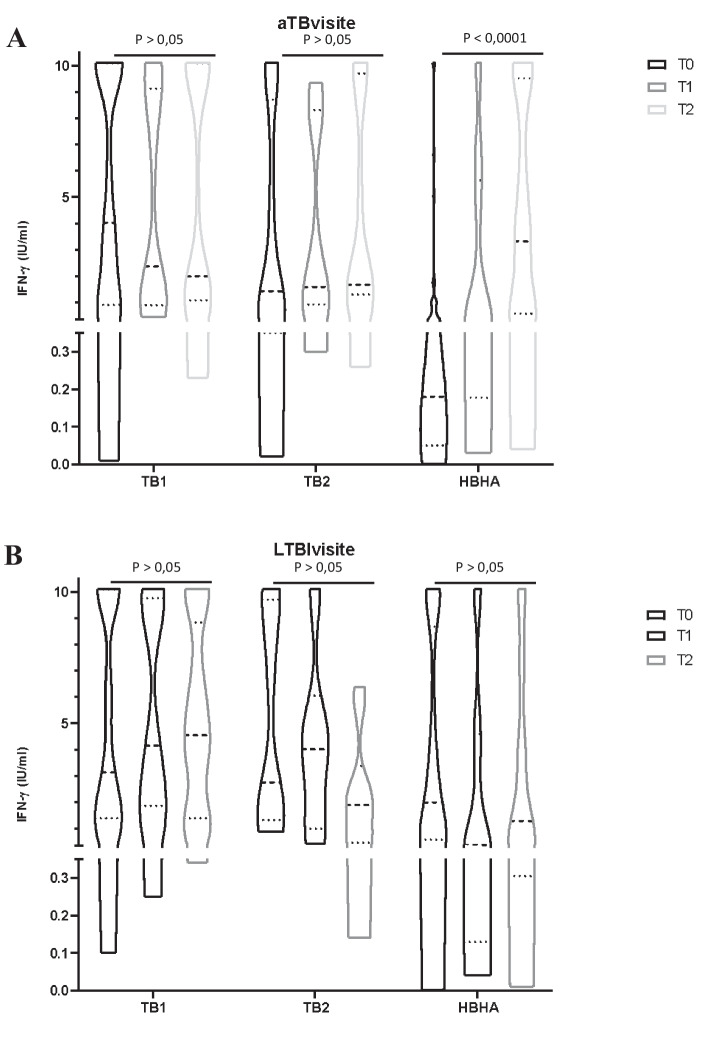
TB1, TB2, and HBHA IFN-gamma responses active TB (**A**) and LTBI (**B**) children evaluated at baseline (T0), after 3 months of therapy (T1) and at the end of the treatment (T2)

Plasma IFN-γ levels in response to TB1, TB2, or HBHA stimulations were measured after 3 months (T1) and at the end of treatment (T2) of LTBI or active TB (Fig. 3).

In children with active TB, the median IFN-γ response to TB1 and TB2 remained similar during the three time points (*p* > 0.05), while the median response to HBHA increased significantly (*p* < 0.0001) (Fig. 3a). Therapy for active disease was associated with increase of HBHA-specific responses in 18 of the 22 children with active TB not responding to HBHA at baseline. The proportion of positive HBHA-based IGRA tests, using the cut-off value of 0.25 IU/ml, increased from T0 (19/43) to T2 (25/28).

Conversely, in LTBI children the IFN-γ levels in theTB1, TB2, and HBHA groups remained similar at all time points (*p* > 0.05, Fig. 3), although treated LTBI patients showed lower IFN-γ values in the TB2 group, that however failed to reach statistical significance.

### Ability to respond to HBHA is maintained in asymptomatic active TB children

**Fig. 4:**
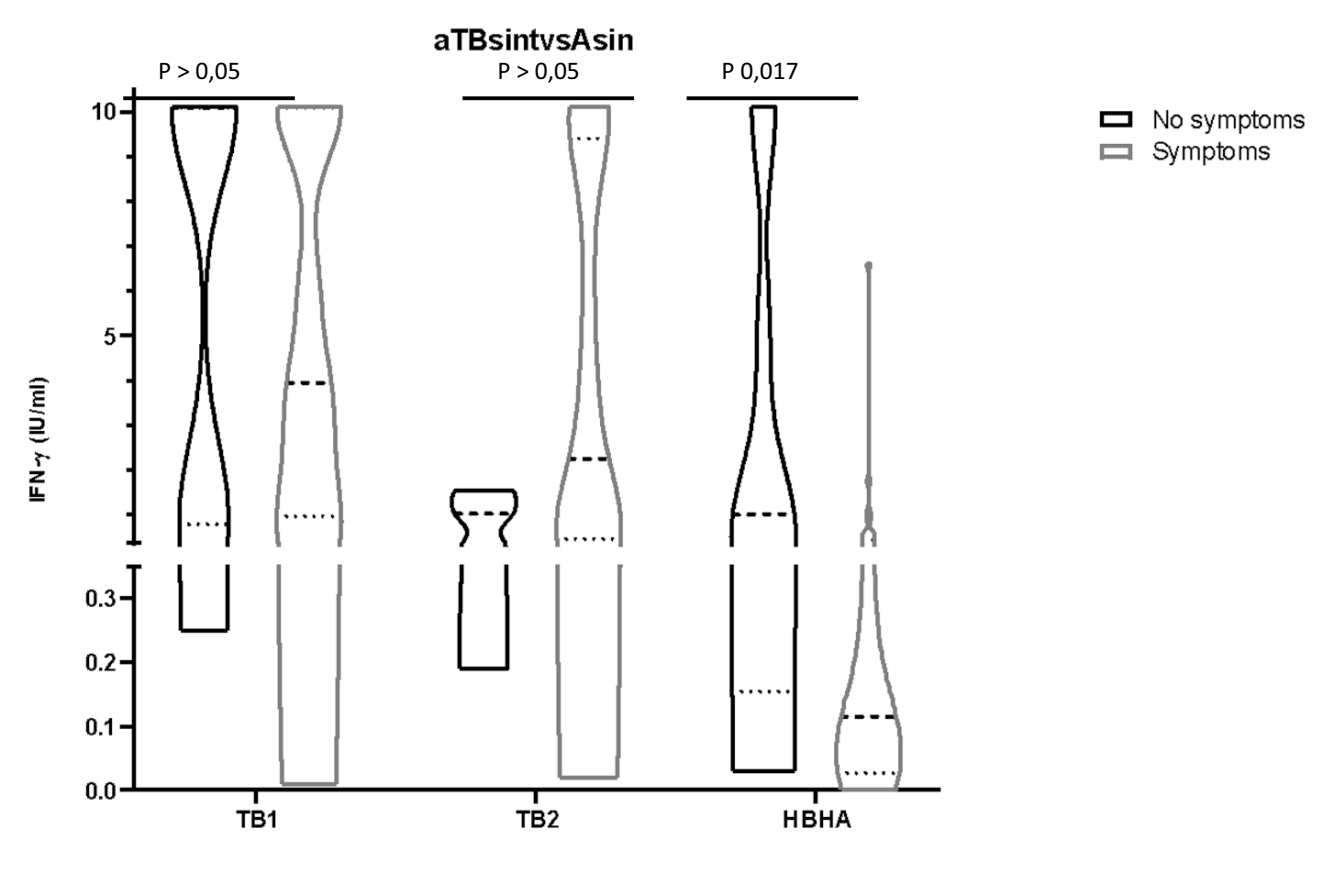
TB1, TB2, and HBHA IFN-gamma responses in children asymptomatic or symptomatic active TB

Among the 44 children diagnosed with active TB, 8 did not show signs or symptoms of disease yet had a positive microbiological result for Mtb infection. These 8 asymptomatic children were evaluated within active case finding because they were contacts of active pulmonary TB patients, and had clinical, radiological, and immunological (QFT) assessment. We compared TB1, TB2, and HBHA responses between the symptomatic and asymptomatic active TB children (Fig. 4). Plasma IFN-γ levels in response to TB1 and TB2 were similar in each group (symptomatic and asymptomatic ATB) and did not present statistically significant differences (*p* > 0.05). Conversely, 6 out of 8 (75%) asymptomatic active TB children were fully capable of responding to HBHA (mean values of IFN-γ 1.01 IU/ml), while 6 out of 36 (16.6%) symptomatic active TB children in vitro responded to HBHA with a mean value of IFN-γ of 0.115 IU/ml (*p* 0.017).

### QFT and HBHA-IGRA quantitative responses in children with active TB change according to disease severity

**Fig. 5:**
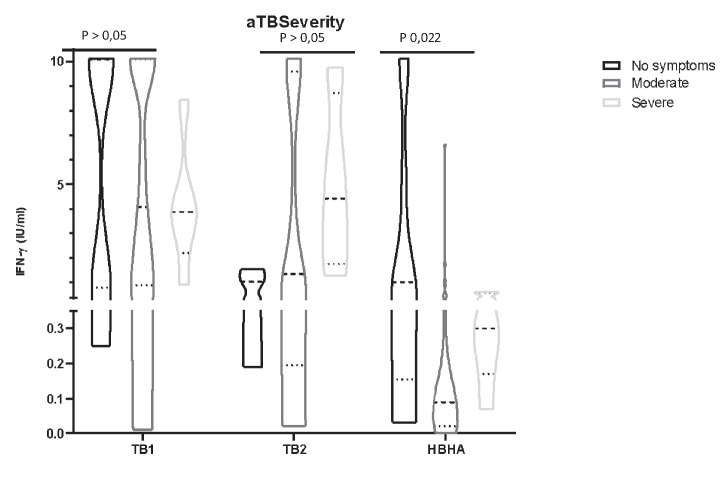
TB1, TB2, and HBHA IFN-gamma responses in children with asymptomatic, moderate, or severe active TB

We compared plasma IFN-γ levels in children with asymptomatic, moderate, and severe active TB (Fig. 5). TB1 and TB2 levels did not significantly change according to disease severity (*p* > 0.05), although severe active TB children had higher mean values in both TB1 and TB2 tubes. In contrast, the median IFN-γ response to HBHA significantly changed in the three severity groups, with asymptomatic active TB children having the highest responses (*p* 0.022).

## Discussion

In our study, we found that an HBHA-based IGRA, when combined with the commercially available QFT-Plus, improved the ability of differentiating different stages of the TB spectrum in children. In particular, children with LTBI or asymptomatic TB were able to respond to HBHA antigens, while those with symptomatic active TB were not. Interestingly, children with active TB that showed clinical and microbiological response to treatment restored the ability to produce IFN-γ when stimulated with HBHA antigen. All together, our study suggests that an HBHA-based IGRA may support clinical practice — along with routine clinical, radiologic, and microbiological data — in differentiating active TB and LTBI in children, or in recognizing those children that are having an appropriate response to medical treatment, which can be particularly useful for those patients with microbiologically negative TB.

During recent years, advances in basic microbiology and immunology and improved diagnostics are starting to shed some light on the complex interplay between the host immune system and Mtb. There is increasing recognition and understanding that the relationships between the human host and *Mtb* are better defined by a complex and dynamic spectrum of conditions (ranging from clearance of Mtb after exposure, to latent infection, incipient and subclinical TB, to different degrees of severity of clinically evident TB) rather than by the classic dichotomous distinction between active TB and LTBI [22–25]. In this regard, pediatric TB perfectly falls within this complex spectrum of consequences of Mtb infection. In fact, while younger children have historically higher risk of progression from infection to severe disease [26], they also have more frequently a paucibacillary disease or subtle clinical presentations, making a definite diagnosis of active TB or LTBI, or even a differential diagnosis with other infectious and non-infectious diseases (e.g., lymphomas), challenging. Biomarkers that support in the understanding of where a child is positioned in the TB spectrum can aid in the diagnostic and therapeutic decisions. Proper classifications are particularly important in children, since child-friendly drug formulations are not available for most drugs [27], multiple drugs for several months are usually needed, and side effects can affect several systems.

During the last few years, several attempts were made to develop new immunological tests capable of discriminating between stages of TB infection [28–30]. However, despite some progress [31], a reliable biomarker with prognostic value is still missing. For example, a host blood transcriptomic RNA-signature (RISK11) was found to recognize active TB in symptomatic patients and to identify Mtb-infected patients at increased risk for progression to TB disease [32]. However, provision of preventive therapy to asymptomatic RISK11 positive patients did not reduce risk of progression to TB [32]. These findings obtained in a large cohort reinforce the need of new tests to support clinical-decision rules that may eventually require the combined use of two or more assays.

The results of our study reinforce our previous preliminary study on a smaller cohort of children with LTBI or active TB tested with QFT-GIT and HBHA-IGRA, showing that QFT-GIT responses were similar in any stage of the TB spectrum [16]). However, the new-generation QFT-Plus, discriminating CD4 + (TB1) and CD8 + (TB2) responses to TB antigens, had the theoretical advantage to better support the immunological characterization of active TB and LTBI, which however has been found to be not highly accurate by recent adult studies [33, 34]. In our new study, including a higher number of LTBI and active TB cases, TB1 and TB2 mean responses were overall overlapping between active TB and LTBI children, suggesting that the QFT-Plus is not providing prognostic support in the immunological diagnosis of TB in children. Conversely, HBHA-IGRA responses significantly differ in these two groups, with LTBI children having higher responses. This scenario suggests that children with LTBI, characterized by a simultaneous strong IFN-γ responses to QFT (regardless of tube) and HBHA-based IGRA, may be distinguished from patients with active TB, subclinical TB or incipient TB, that start losing the ability to respond to HBHA while maintaining a strong response against RD1 antigens.

Our findings are in line with two recent, independent studies that assessed a combined QFT-Plus and HBHA signature in adults [35, 36]. Chedid et al. performed a 3-year prospective multicenter study evaluating 132 adults with culture-confirmed TB, in high TB burden areas in Middle-East Asiatic areas [35]. They found that HBHA baseline responses were low in majority of cases and significantly increased during treatment. Importantly, while the accuracy of the QFT-P and rmsHBHA IGRAs compared to culture throughout treatment was low (40 and 65% respectively), combining both tests improved their sensitivity and accuracy (70–80%), supporting the theory that the complexity of TB pathogenesis mainly requires a combination of biomarkers for its proper characterization, rather than focusing on single biomarkers [37]. Similarly, Tang et al. studied 135 healthcare workers (HCWs) and 57 patients with active pulmonary TB in a Chinese TB Hospital [38]. In order to assess the specific contribution of TB1 and TB2 tubes in discriminating active TB and LTBI, they measured the differences of the quantitative TB responses in the two tubes (QFT-Plus TB2 less TB1). They found that a comparison of TB2 and TB1 responses (QFT-Plus TB2 less TB1, which was positively correlated with CD8 T-cell response—*r* = 0.731, *p* < 0.001) was higher in active TB cases, while the HBHA-induced IFN-γ response was significantly higher in the LTBI (median 69.67 pg/ml; both *p* < 0.0001). Importantly, also in their cohort, after combining the HBHA-IGRA with QFT-Plus results, the accuracy of identifying ATB and LTBI was improved to 85.7% from 74.3%.

The restoration of the HBHA-induced IFN-γ response occurring during effective TB treatment, confirmed in adults and pediatric studies [16, 35, 36], has potential significant application in clinical practice. Traditional guidelines state that a 9-month regimen of isoniazid can prevent active TB in persons with LTBI and that a pulmonary TB should be treated for 6 months [39]. However, modern medicine is shifting toward shorter or personalized treatment regimens for several bacterial infections, including pneumonia [40, 41]. Recently, two major studies showed that shorter regimens may be used in TB patients as well. Adult studies demonstrated that a 4-month regimen of rifampin was not inferior to the 9-month regimen of isoniazid for the prevention of active TB in adults and was associated with a higher rate of treatment completion and better safety [42]. Similarly, in active TB adults the efficacy of a 4-month rifapentine-based regimen containing moxifloxacin was noninferior to the standard 6-month regimen in the treatment of TB [43]. While these studies open a new era for TB treatment, since shorter regimens can significantly improve adherence and reduce side effects, the use of reliable biomarkers may support patients’ stratification, identifying those that may benefit from shorter regimens. In bacterial infections, monitoring C-reactive protein or procalcitonine in blood can be useful to support the decision of early stop of antibiotics [40, 41]. In TB, biomarkers such as HBHA-based IGRAs can identify those patients whose immune system is already showing the ability to contain, at least partially, the disease and these subsets of people may significantly benefit from shorter regimens. Similarly, monitoring TB treatments with HBHA-based IGRAs may aid in the tuning of the regimen. Such biomarkers can be particularly useful considering new findings that also in pediatric non-severe TB shorter regimens may be effective [44]. The next phase of shortened TB treatments would be to assess how biomarker-oriented regimens may offer or not benefit in personalized treatment strategies. Interestingly, children treated with LTBI presented a drop in the ability to produce IFN-γ in the TB2 tube, which accounts for the CD8 + responses. Considering that CD8 + responses have been linked with active TB with bacillary disease [45], the drop we found can suggest a successful clearance of the latent bacilli.

**Fig. 6:**
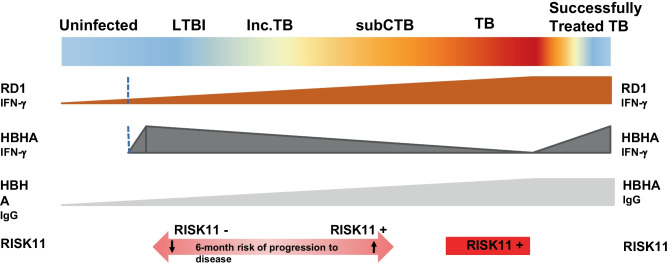
Proposal of a multi-parameter biomarker approach for the definition of the TB spectrum according to recent studies. Inc.TB, incipient TB; subCTB, subclinical active TB or asymptomatic active TB

In view of the findings of our study, which further confirm previous pediatric and adult studies on the role of HBHA-IGRA in defining the TB spectrum [14–16], and the advances obtained by new transcriptomic technologies in recognizing symptomatic patients or those at highest risk of progression to disease within the following 6 months (RISK-11) [32], we may expect in a near future that the combined use of biomarkers (including HBHA-based IGRAs) may be useful to better define the different TB stages, providing prognostic support to the currently used tests (Fig. 6). Specifically, such an approach would be particularly appropriate in low TB incidence countries, where the 6-month performance of RISK11 would be more useful given the theorical lower risk of new TB infections in patients with a RISK11 negative test. In fact, the poor performance of RISK11 after the 6 months in the cohort of South African adults may be due to new infections rather than false negative RISK11 tests [32].

Our study presents some limitations. The overall low number of children with active TB or LTBI is a limitation of this study. The study has been performed in a low-TB incidence area and the HBHA-assay is not yet easily available in all settings. Also, not all children were assessed at all time points, mainly due to logistic difficulties of some families to be reassessed at predefined timings. Also, the first cohort of children was tested with QFT-GIT, which only offers TB1 (CD4 +) responses. We could not calculate the sensitivity, specificity, positive predictive value, and negative predictive value of the HBHA-IGRA as it was only tested in children that were diagnosed with LTBI or active TB, according to clinical, microbiological, and radiologic data. Another problem in calculating these parameters in active and in LTBI patients is the lack of a gold standard for the diagnosis of LTBI. For these reasons, we decided to compare quantitative IGRA and HBHA responses in different TB groups showing that IGRAs have mostly similar responses in all subgroups and HBHA can add a further discrimination, and this is why we conclude that a multiparameter approach, as proposed in Fig. 6, can help in understanding the complexity of the TB spectrum in children. Last, some IGRA data were lacking at different time points because patients either missed a control or some of them had not reached yet the specific follow-up point since diagnosis. Despite the mentioned limitations, this study still represents the first prospective assessment in children with a wide spectrum of TB manifestations of well-recognized biomarkers from available literature with a defined pathophysiological role in TB pathogenesis.

In conclusion, our results suggest that an HBHA-based IGRA, combined with the commercially available IGRAs, may provide a useful tool to help pediatricians to shed some light on the features of TB spectrum in children, supporting the diagnosis of LTBI or active TB, to define the less severe forms of active TB and to monitor treatment response, particularly in children with microbiologically non-confirmed active TB, for which to document culture conversions is not possible. Studies on independent cohorts and including a larger number of patients are needed to confirm our findings and understand how this biomarker can help in the personalized management of TB in children.

## Data Availability

Available upon request to the corresponding author.

## Abbreviations

IGRA: Interferon-gamma releasing assay
HBHA: Heparin-binding haemagglutinin
TB: Tuberculosis
WHO: World Health Organization
TST: Tuberculin skin test
QFT-GIT: QuantiFERON-TB Gold in Tube

## References

[1] Newton SM, Brent AJ, Anderson S, Whittaker E, Kampmann B (2008). Paediatric tuberculosis. Lancet Infect Dis.

[2] Global tuberculosis report (2020) Geneva: World Health Organization. Licence: CC BY-NC-SA 3.0 IGO

[3] Migliori GB, Thong PM, Akkerman O, Alffenaar JW, Álvarez-Navascués F, Assao-Neino MM, Bernard PV, Biala JS, Blanc FX, Bogorodskaya EM, Borisov S, Buonsenso D, Calnan M, Castellotti PF, Centis R, Chakaya JM, Cho JG, Codecasa LR, D'Ambrosio L, Denholm J, Enwerem M, Ferrarese M, Galvão T, García-Clemente M, García-García JM, Gualano G, Gullón-Blanco JA, Inwentarz S, Ippolito G, Kunst H, Maryandyshev A, Melazzini M, de Queiroz Mello FC, Muñoz-Torrico M, Njungfiyini PB, Palmero DJ, Palmieri F, Piccioni P, Piubello A, Rendon A, Sabriá J, Saporiti M, Scognamiglio P, Sharma S, Silva DR, Souleymane MB, Spanevello A, Tabernero E, Tadolini M, Tchangou ME, Thornton ABY, Tiberi S, Udwadia ZF, Sotgiu G, Ong CWM, Goletti D (2020) Worldwide effects of coronavirus disease pandemic on tuberculosis services, January-April 2020. Emerg Infect Dis 26(11):2709–2712. 10.3201/eid2611.203163. Epub 2020 Sep 11 PMID: 32917293; PMCID: PMC7588533 10.3201/eid2611.203163 PMC7588533 32917293

[4] Buonsenso D, Iodice F, Sorba Biala J, Goletti D (2021) COVID-19 effects on tuberculosis care in Sierra Leone. Pulmonology 27(1):67–69. 10.1016/j.pulmoe.2020.05.013. Epub 2020 Jun 6 PMID: 32561353; PMCID: PMC7275172 10.1016/j.pulmoe.2020.05.013 PMC7275172 32561353

[5] Mandal N, Anand PK, Gautam S, Das S, Hussain T (2017). Diagnosis and treatment of paediatric tuberculosis: an insight review. Crit Rev Microbiol.

[6] Campbell JR, Dowdy D, Schwartzman K (2019). Treatment of latent infection to achieve tuberculosis elimination in low-incidence countries. PLoS Med.

[7] Churchyard GJ, Fielding KL, Lewis JJ, Coetzee L, Corbett EL, Godfrey-Faussett P (2014). A trial of mass isoniazid preventive therapy for tuberculosis control. N Engl J Med.

[8] Sali M, Buonsenso D, Goletti D, D’Alfonso P, Zumbo A, Fadda G, Sanguinetti M, Delogu G, Valentini P (2015). Accuracy of QuantiFERON-TB Gold test for tuberculosis diagnosis in children. PLoS ONE.

[9] Amicosante M, D'Ambrosio L, Munoz M, Mello FCQ, Tebruegge M, Chegou NN et al (2017) TB Diagnostic Survey Working Group. Current use and acceptability of novel diagnostic tests for active tuberculosis: a worldwide survey. J Bras Pneumol 43:380–392 10.1590/S1806-37562017000000219 PMC5790656 29160384

[10] QuantiFERON-TB Gold Plus package insert. Available at: https://www.quantiferon.com/products/quantiferon-tb-gold-plus-qft-plus/ . Accessed 18 Oct 2021.

[11] Buonsenso D, Delogu G, Perricone C, Grossi R, Careddu A, De Maio F, Palucci I, Sanguinetti M, Valentini P, Sali M (2020). Accuracy of QuantiFERON-TB Gold Plus Test for diagnosis of Mycobacterium tuberculosis infection in children. J Clin Microbiol.

[12] Soler-Garcia A, Gamell A, Santiago B, Monsonís M, Cobo-Vázquez E, Bustillo-Alonso M, Tagarro A, Pérez-Gorricho B, Espiau M, Piqueras AI, Korta-Murua JJ, Rodríguez-Molino P, Lobato Z, Pérez-Porcuna T, Tebruegge M, Noguera-Julian A (2021) QFT-Plus Study Group of the Spanish Pediatric TB Research Network (pTBred). Quantiferon-TB Gold Plus assay specificity in children and adolescents with suspected tuberculosis-a multicenter cross-sectional study in Spain. Pediatr Infect Dis J 22. 10.1097/INF.0000000000003173. Epub ahead of print PMID: 34310504 10.1097/INF.0000000000003173 34310504

[13] Nguyen DT, Phan H, Trinh T, Nguyen H, Doan H, Pham N, Nguyen H, Nguyen H, Nguyen HV, Le HV, Nguyen N, Graviss EA (2019). Sensitivity and characteristics associated with positive QuantiFERON-TB Gold-Plus assay in children with confirmed tuberculosis. PLoS ONE.

[14] De Maio F, Squeglia F, Goletti D, Delogu G (2019). The mycobacterial HBHA protein: a promising biomarker for tuberculosis. Curr Med Chem.

[15] Goletti D, Delogu G, Matteelli A, Migliori GB (2022). The role of IGRA in the diagnosis of tuberculosis infection, differentiating from active tuberculosis, and decision making for initiating treatment or preventive therapy of tuberculosis infection. Int J Infect Dis.

[16] Sali M, Buonsenso D, D'Alfonso P, De Maio F, Ceccarelli M, Battah B, Palucci I, Chiacchio T, Goletti D, Sanguinetti M, Valentini P, Delogu G (2018). Combined use of Quantiferon and HBHA-based IGRA supports tuberculosis diagnosis and therapy management in children. J Infect.

[17] Perez-Velez CM, Marais BJ (2012). Tuberculosis in children. N Engl J Med.

[18] Tebruegge M, Salo E, Ritz N, Kampmann B (2013) The Paediatric Tuberculosis Network European Trialsgroup Ptbnet. Inclusion of latent tuberculosis infection as a separate entity into the international classification of diseases. Thorax 68(6):588. 10.1136/thoraxjnl-2012-202824. Epub 2012 Nov . PMID: 23128034 10.1136/thoraxjnl-2012-202824 23128034

[19] Buonsenso D, Lancella L, Delogu G, Krzysztofiak A, Testa A, Ranno O, D’Alfonso P, Valentini P (2012). A twenty-year retrospective study of pediatric tuberculosis in two tertiary hospitals in Rome. Pediatr Infect Dis J.

[20] Delogu G, Chiacchio T, Vanini V, Butera O, Cuzzi G, Bua A, Molicotti P, Zanetti S, Lauria FN, Grisetti S, Magnavita N, Fadda G, Girardi E, Goletti D (2011) Methylated HBHA produced in M. smegmatis discriminates between active and non-active tuberculosis disease among RD1-responders. PLoS One 29;6(3):e18315. 10.1371/journal.pone.0018315. PMID: 21479248; PMCID: PMC3066236 10.1371/journal.pone.0018315 PMC3066236 21479248

[21] Delogu G, Bua A, Pusceddu C, Parra M, Fadda G, Brennan MJ (2004). Expression and purification of recombinant methylated HBHA in Mycobacterium smegmatis. FEMS Microbiol Lett.

[22] Barry CE, Boshoff HI, Dartois V (2009). The spectrum of latent tuberculosis: rethinking the biology and intervention strategies. Nat Rev Microbiol.

[23] Delogu G, Goletti D (2014). The spectrum of tuberculosis infection: new perspectives in the era of biologics. J Rheumatol Suppl.

[24] Buonsenso D, Sali M, Focarelli B, Onesimo R, Palucci I, Delogu G, Valentini P (2020) The tuberculosis spectrum: translating basic research into pediatric clinical practice. Med Hypotheses 141:108091. 10.1016/j.mehy.2015.10.028. Epub 2015 Oct 28 PMID: 26547272 10.1016/j.mehy.2015.10.028 26547272

[25] Pai M, Behr MA, Dowdy D, Dheda K, Divangahi M, Boehme CC, Ginsberg A, Swaminathan S, Spigelman M, Getahun H, Menzies D, Raviglione M (2016). Tuberculosis Nat Rev Dis Primers.

[26] Basu Roy R, Thee S, Blázquez-Gamero D, Falcón-Neyra L, Neth O, Noguera-Julian A, Lillo C, Galli L, Venturini E, Buonsenso D, Götzinger F, Martinez-Alier N, Velizarova S, Brinkmann F, Welch SB, Tsolia M, Santiago-Garcia B, Krüger R, Tebruegge M (2020) ptbnet TB Meningitis Study Group. Performance of immune-based and microbiological tests in children with tuberculosis meningitis in Europe: a multicentre Paediatric Tuberculosis Network European Trials Group (ptbnet) study. Eur Respir J 56(1):1902004. 10.1183/13993003.02004-2019. PMID: 32299859; PMCID: PMC7330130 10.1183/13993003.02004-2019 PMC7330130 32299859

[27] Noguera-Julian A, Buonsenso D, McKenna L, Seddon JA, Ritz N (2021). Availability of fixed-dose, child-friendly formulations of first-line tuberculosis drugs in Europe. Eur Respir J.

[28] Garay-Baquero DJ, White CH, Walker NF, Tebruegge M, Schiff HF, Ugarte-Gil C, Morris-Jones S, Marshall BG, Manousopoulou A, Adamson J, Vallejo AF, Bielecka MK, Wilkinson RJ, Tezera LB, Woelk CH, Garbis SD, Elkington P (2020) Comprehensive plasma proteomic profiling reveals biomarkers for active tuberculosis. JCI Insight. 5(18):e137427. 10.1172/jci.insight.137427. PMID: 32780727; PMCID: PMC7526553 10.1172/jci.insight.137427 PMC7526553 32780727

[29] Villanueva P, Sudbury E, Song R, Tebruegge M, Curtis N (2019). Advanced immunodiagnostic tests for paediatric tuberculosis. Lancet Infect Dis.

[30] Tebruegge M, Ritz N, Donath S, Dutta B, Forbes B, Clifford V, Zufferey C, De Rose R, Robins-Browne RM, Hanekom W, Graham SM, Connell T, Curtis N (2019). Mycobacteria-specific mono- and polyfunctional CD4+ T cell profiles in children with latent and active tuberculosis: a prospective proof-of-concept study. Front Immunol.

[31] Sudbury EL, Clifford V, Messina NL, Song R, Curtis N (2020). Mycobacterium tuberculosis-specific cytokine biomarkers to differentiate active TB and LTBI: A systematic review. J Infect.

[32] Scriba TJ, Fiore-Gartland A, Penn-Nicholson A, Mulenga H, Kimbung Mbandi S, Borate B, Mendelsohn SC, Hadley K, Hikuam C, Kaskar M, Musvosvi M, Bilek N, Self S, Sumner T, White RG, Erasmus M, Jaxa L, Raphela R, Innes C, Brumskine W, Hiemstra A, Malherbe ST, Hassan-Moosa R, Tameris M, Walzl G, Naidoo K, Churchyard G, Hatherill M(2021) CORTIS-01 Study Team. Biomarker-guided tuberculosis preventive therapy (CORTIS): a randomised controlled trial. Lancet Infect Dis 21(3):354–365. 10.1016/S1473-3099(20)30914-2 10.1016/S1473-3099(20)30914-2 PMC7907670 33508224

[33] Petruccioli E, Vanini V, Chiacchio T, Cirillo DM, Palmieri F, Ippolito G, Goletti D (2016). Modulation of interferon-gamma response to QuantiFERON-TB-plus detected by enzyme-linked immunosorbent assay in patients with active and latent tuberculosis infection. Int J Mycobacteriol.

[34] Petruccioli E, Vanini V, Chiacchio T, Cuzzi G, Cirillo DM, Palmieri F, Ippolito G, Goletti D (2017). Analytical evaluation of QuantiFERON- Plus and QuantiFERON- Gold In-tube assays in subjects with or without tuberculosis. Tuberculosis (Edinb).

[35] Chedid C, Kokhreidze E, Tukvadze N, Banu S, Uddin MKM, Biswas S, Russomando G, Acosta CCD, Arenas R, Ranaivomanana PP, Razafimahatratra C, Herindrainy P, Rakotonirina J, Raherinandrasana AH, Rakotosamimanana N, Hamze M, Ismail MB, Bayaa R, Berland JL, De Maio F, Delogu G, Endtz H, Ader F, Goletti D, Hoffmann J (2021) Relevance of QuantiFERON-TB Gold Plus and Heparin-binding hemagglutinin Interferon-γ release assays for monitoring of pulmonary tuberculosis clearance: a multicentered study. Front Immunol 11:616450. 10.3389/fimmu.2020.616450. PMID: 33603746; PMCID: PMC7885528 10.3389/fimmu.2020.616450 PMC7885528 33603746

[36] Tang J, Huang Y, Jiang S, Huang F, Ma T, Qi Y, Ma Y (2020) QuantiFERON-TB Gold Plus combined with HBHA-Induced IFN-γ release assay improves the accuracy of identifying tuberculosis disease status. Tuberculosis (Edinb) 124:101966. 10.1016/j.tube.2020.101966. Epub 2020 Aug 6 PMID: 32866942 10.1016/j.tube.2020.101966 32866942

[37] Sudbury EL, Otero L, Tebruegge M, Messina NL, Seas C, Montes M, Rìos J, Germano S, Gardiner K, Clifford V, Gotuzzo E, Curtis N (2019) Mycobacterium tuberculosis-specific cytokine biomarkers for the diagnosis of childhood TB in a TB-endemic setting. J Clin Tuberc Other Mycobact Dis 16:100102. 10.1016/j.jctube.2019.100102. PMID: 31720428; PMCID: PMC6830137 10.1016/j.jctube.2019.100102 PMC6830137 31720428

[38] Tang J, Huang Y, Jiang S, Huang F, Ma T, Qi Y, Ma Y (2020) QuantiFERON-TB Gold Plus combined with HBHA-Induced IFN-γ release assay improves the accuracy of identifying tuberculosis disease status. Tuberculosis (Edinb) 124:101966. 10.1016/j.tube.2020.101966. Epub 2020 Aug 6 PMID: 32866942 10.1016/j.tube.2020.101966 32866942

[39] WHO. Guidance for national tuberculosis programmes on the management of tuberculosis in children. https://www.who.int/publications/i/item/9789241548748

[40] Bielicki JA, Stöhr W, Barratt S, Dunn D, Naufal N, Roland D, Sturgeon K, Finn A, Rodriguez-Ruiz JP, Malhotra-Kumar S, Powell C, Faust SN, Alcock AE, Hall D, Robinson G, Hawcutt DB, Lyttle MD, Gibb DM, Sharland M (2021) PERUKI, GAPRUKI, and the CAP-IT Trial Group. Effect of amoxicillin dose and treatment duration on the need for antibiotic re-treatment in children with community-acquired pneumonia: the CAP-IT randomized clinical trial. JAMA 326(17):1713–1724. 10.1001/jama.2021.17843. PMID: 34726708; PMCID: PMC8564579 10.1001/jama.2021.17843 PMC8564579 34726708

[41] Buonsenso D, De Rose C (2022) Implementation of lung ultrasound in low- to middle-income countries: a new challenge global health? Eur J Pediatr 181(1):1–8. 10.1007/s00431-021-04179-9. Epub 2021 Jul 3 PMID: 34216270; PMCID: PMC8254441 10.1007/s00431-021-04179-9 PMC8254441 34216270

[42] Menzies D, Adjobimey M, Ruslami R, Trajman A, Sow O, Kim H, Obeng Baah J, Marks GB, Long R, Hoeppner V, Elwood K, Al-Jahdali H, Gninafon M, Apriani L, Koesoemadinata RC, Kritski A, Rolla V, Bah B, Camara A, Boakye I, Cook VJ, Goldberg H, Valiquette C, Hornby K, Dion MJ, Li PZ, Hill PC, Schwartzman K, Benedetti A (2018). Four months of rifampin or nine months of isoniazid for latent tuberculosis in adults. N Engl J Med.

[43] Dorman SE, Nahid P, Kurbatova EV, Phillips PPJ, Bryant K, Dooley KE, Engle M, Goldberg SV, Phan HTT, Hakim J, Johnson JL, Lourens M, Martinson NA, Muzanyi G, Narunsky K, Nerette S, Nguyen NV, Pham TH, Pierre S, Purfield AE, Samaneka W, Savic RM, Sanne I, Scott NA, Shenje J, Sizemore E, Vernon A, Waja Z, Weiner M, Swindells S, Chaisson RE (2021) AIDS Clinical Trials Group; Tuberculosis Trials Consortium. Four-Month Rifapentine Regimens with or without Moxifloxacin for Tuberculosis. N Engl J Med 384(18):1705–1718. 10.1056/NEJMoa2033400. PMID: 33951360; PMCID: PMC8282329 10.1056/NEJMoa2033400 PMC8282329 33951360

[44] Turkova A, Wills GH, Wobudeya E, Chabala C, Palmer M, Kinikar A, Hissar S, Choo L, Musoke P, Mulenga V, Mave V, Joseph B, LeBeau K, Thomason MJ, Mboizi RB, Kapasa M, van der Zalm MM, Raichur P, Bhavani PK, McIlleron H, Demers AM, Aarnoutse R, Love-Koh J, Seddon JA, Welch SB, Graham SM, Hesseling AC, Gibb DM, Crook AM (2022) SHINE Trial Team. Shorter treatment for nonsevere tuberculosis in African and Indian children. N Engl J Med 386(10):911–922. 10.1056/NEJMoa2104535. PMID: 35263517 10.1056/NEJMoa2104535 PMC7612496 35263517

[45] Petruccioli E, Chiacchio T, Pepponi I, Vanini V, Gualano G, Cuzzi G, Cirillo D, Palmieri F, Goletti D (2016) CD8-response associates with active tuberculosis and TB2-tube response in the QuantiFERON-TB-Plus kit. European Respir J 48(suppl 60):PA2693. 10.1183/13993003.congress-2016.PA2693

